# Comparative speed of kill provided by lotilaner (Credelio™), sarolaner (Simparica Trio™), and afoxolaner (NexGard™) to control *Amblyomma americanum* infestations on dogs

**DOI:** 10.1186/s13071-024-06363-w

**Published:** 2024-07-20

**Authors:** Kathryn E. Reif, Todd M. Kollasch, Jacqueline C. Neilson, Brian H. Herrin, William G. Ryan, Marjorie C. Bell, Mallory S. Beltz, Michael W. Dryden, Jeba R. J. Jesudoss Chelladurai, Kamilyah R. Miller, Cameron J. Sutherland

**Affiliations:** 1 Department of Pathobiology, College of Veterinary Medicine, Auburn University, Auburn, AL USA; 2 Elanco Animal Health Inc, 2500 Innovation Way, Greenfield, IN USA; 3 Department of Diagnostic Medicine/Pathobiology, College of Veterinary Medicine, Kansas State University, Manhattan, KS USA; 4 Ryan Mitchell Associates LLC, 16 Stoneleigh Park, Westfield, NJ USA; 5 Fort Collins, CO USA

**Keywords:** Afoxolaner, *Amblyomma americanum*, Canine, Credelio, Dog, Lone star tick, Lotilaner, NexGard, Sarolaner, Simparica Trio

## Abstract

**Background:**

Canine acaricides with rapid onset and sustained activity can reduce pathogen transmission risk and enhance pet owner experience. This randomized, complete block design, investigator-masked study compared the speed of kill of *Amblyomma americanum* provided by three monthly-use isoxazoline-containing products.

**Methods:**

Eight randomized beagles per group were treated (day 0), per label, with sarolaner (combined with moxidectin and pyrantel, Simparica Trio™), afoxolaner (NexGard™), or lotilaner (Credelio™), or remained untreated. Infestations with 50 adult *A. americanum* were conducted on days − 7, − 2, 21, and 28, and tick counts were performed on day − 5 (for blocking), and at 4, 8, 12, 24, 48, and 72 h following treatment and subsequent infestations. Efficacy calculations were based on geometric mean live tick counts. A linear mixed model was used for between-group comparisons.

**Results:**

On day 0, only lotilaner significantly reduced an *A. americanum* infestation by 12 h (43.3%; *P* = 0.002). Efficacy of lotilaner and afoxolaner at 24 h post-treatment was 95.3% and 97.6%, respectively, both significantly different from sarolaner (74%) (*P* = 0.002, *P* < 0.001, respectively). On day 21, at 12 h postinfestation, lotilaner efficacy (59.6%) was significantly different from sarolaner (0.0%) (*P* < 0.001) and afoxolaner (6.3%) (*P* < 0.001). At 24 h, lotilaner efficacy (97.4%) was significantly different (*P* < 0.001) from sarolaner and afoxolaner (13.6% and 14.9%, respectively). On day 28, at 12 h postinfestation, lotilaner efficacy (47.8%) was significantly different from sarolaner (17.1%) (*P* = 0.020) and afoxolaner (9.0%) (*P* = 0.006). At 24 h, lotilaner efficacy (92.3%) was significantly different from sarolaner 4.9% (*P* < 0.001) and afoxolaner (0.0%) (*P* < 0.001). Speed of kill for sarolaner and afoxolaner, but not lotilaner, significantly declined over the study period. Following reinfestation on day 28, neither sarolaner nor afoxolaner reached 90% efficacy by 48 h. By 72 h, sarolaner efficacy was 97.4% and afoxolaner efficacy was 86.3%. Only lotilaner achieved ≥ 90% efficacy by 24 h post-treatment and 24 h postinfestation on days 21 and 28. Time to ≥ 90% efficacy following new infestations consistently occurred 24–48 h earlier for lotilaner compared with sarolaner or afoxolaner.

**Conclusions:**

Credelio (lotilaner) has a more rapid onset of acaricidal activity against *A. americanum* than Simparica Trio (sarolaner-moxidectin-pyrantel) and NexGard (afoxolaner). Only lotilaner’s speed of tick kill is sustained throughout the dosing period.

**Graphical Abstract:**

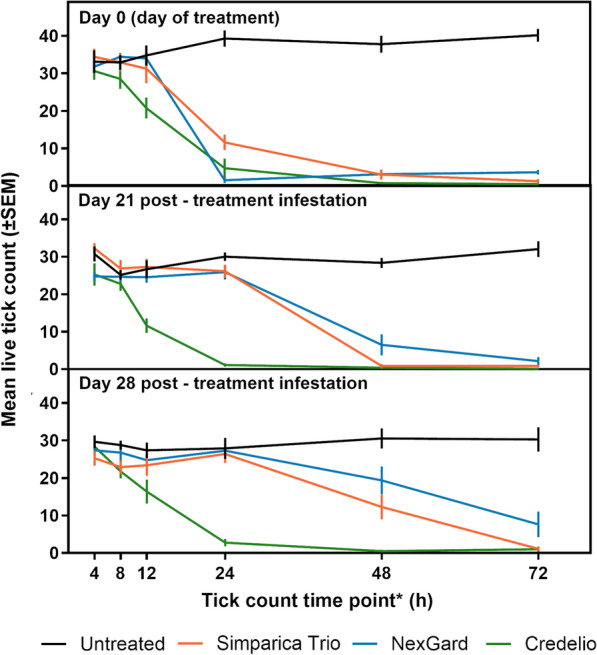

**Supplementary Information:**

The online version contains supplementary material available at 10.1186/s13071-024-06363-w.

## Background

The increasing number of reports from North America of human and canine diseases attributed to tick-transmitted infections aligns with the geographic expansion of ticks, particularly *Ixodes scapularis* and *Amblyomma americanum* [1–6]. Proposed reasons for that expansion include climate change, human behavior, reforestation, population movement, and resurgent host populations, including mammalian and avian wildlife, particularly white-tailed deer [7, 8]. The broadening geographic range of ticks presents a growing threat to companion animals, livestock, and humans.

Reinforcing that threat, a recent paper in the New England Journal of Medicine highlighted the increasing importance of *A. americanum*, a vector of pathogens of human and animal importance including *Francisella tularensis*, *Ehrlichia* spp., and spotted fever group *Rickettsia* spp. [9]. *Amblyomma americanum* is also reported as the second most common tick species infesting cats in the USA and has been demonstrated as an efficient vector of *Cytauxzoon felis* [10–12]. An aggressive hunter tick, *A. americanum*, can rapidly ambulate over many yards upon sensing persistent host odors [8]. Believed in the early twentieth century to be limited to the southeastern USA with a northern geographic limit of southern New Jersey, *A. americanum* has now expanded its range northward through Pennsylvania, New York, and the northeastern Atlantic states into Ontario and Quebec in southern Canada [7, 13]. *Amblyomma americanum* is established in central and mid-western states in the USA, including Kansas and Indiana, where it is the most commonly identified tick, as well as in Michigan, Missouri, Arkansas, Nebraska, Oklahoma, and South Dakota [14–17]. Phenology studies and local tick surveys have demonstrated *A. americanum* adults and nymphs are active early spring through fall, while larvae are most commonly active in the fall [18]. However, when environmental conditions are favorable, host-seeking *A. americanum* may be collected during winter months as well, highlighting its year-round threat [14].

As the geographic distribution of *A. americanum* overlaps with multiple other tick species of veterinary and medical importance, with at least one tick species being active at any given point during the year, prevention and control of canine tick infestations is becoming increasingly important. The more quickly a product acts to kill ticks, the lower the probability that pathogens will be passed from the tick to its host. Among tick-borne bacterial pathogens, transmission can occur within ‘hours to days’ of tick attachment. For example, transmission of *Borrelia burgdorferi* can occur within 24–48 h of attachment of *Ixodes* spp. ticks [19, 20].Rickettsial pathogens can be transmitted even more quickly, with studies demonstrating that *Rickettsia rickettsii* transmission by *Dermacentor variabilis* can occur within 5–20 h after attachment, and by *Amblyomma aureolatum* within 10 h of attachment [21, 22]. Transmission of *Anaplasma phagocytophilum* is more efficient after 36 h of tick attachment, but can occur between 16–24 h [23, 24]. Another study demonstrated that *Ehrlichia canis* (South African strain) can be transmitted by *Rhipicephalus sanguineus* (European strain) within 3–6 h of tick infestation [25]; however, transmission timing of any USA-derived *Ehrlichia* spp. by a given USA-derived tick cohort has not been performed. Compared with tick-borne bacterial pathogens, tick-borne protozoal pathogens are usually transmitted more slowly, within ‘days’ of tick attachment (e.g., *C. felis* transmission by *A. americanum* takes > 36 h), while tick-borne viral pathogens are usually transmitted more quickly, within ‘minutes to hours’ of tick attachment (e.g., Powassan virus transmission by *I. scapularis* from 15 min after attachment) [26, 27]. Transmission timing can also be influenced by interrupted tick feeding, where a tick with its feeding interrupted on one host may transmit a pathogen more quickly upon finding and feeding on a second host ([28], reviewed in [29]). Although different tick-borne pathogens may be transmitted at different speeds and numerous variables may further influence pathogen transmission timing, the longer an infected tick is alive and feeding on a host, the greater the risk of tick-borne pathogen transmission to that host. Thus, attributes of an effective tick control product include a rapid onset of action to provide a quick tick kill, and reliable sustained efficacy throughout the duration of the label indication.

Within the last decade, four isoxazoline compounds, afoxolaner, fluralaner, sarolaner, and lotilaner, have been approved for use in dogs for the treatment and control of *A. americanum*, a challenging tick species for acaricides. Evidence of the relative hardiness of *A. americanum*, compared with other tick species, is reflected on some product labels. For example, fluralaner is approved to provide 8 weeks control of *A. americanum*, compared with 12 weeks for other tick species. For fluralaner, for the combination of sarolaner–moxidectin–pyrantel, and for afoxolaner as a single entity and in combination with moxidectin and pyrantel, the *A. americanum* claim is based on efficacy determined at 72 h post-treatment and 72 h after reinfestation, rather than at the 48-h timepoint used for other labeled tick species [30–32]. However, as pathogen transmission may occur within 24 h of attachment and as studies have demonstrated within-isoxazoline family variation in the speed with which ticks are killed, an understanding of the tick killing efficacy of isoxazoline-containing products at earlier timepoints is important [33, 34].

The objective of the study reported here was to compare the relative speeds of kill of adult *A. americanum* provided by commercially available formulations of three, monthly-dosed isoxazolines: lotilaner (Credelio), sarolaner (in combination with moxidectin and pyrantel, Simparica Trio) and afoxolaner (NexGard), over 1 month following a single treatment, administered per label instructions.

## Methods

### Animals

An acclimation period for 38 beagle dogs began on day 19. For study inclusion, dogs had to be clinically healthy, behaviorally amenable, and have demonstrated susceptibility to tick infestations, based on retention of ≥ 25% of an *A. americanum* test challenge on day − 7. Exclusion criteria included: previous exposure to ticks, treatment with an isoxazoline product in the 12 months prior to the study, treatment with any topically applied acaricide/insecticide (including an acaricidal/insecticidal collar) within 60 days, the presence of circulating antibodies to *Borrelia* spp., *Ehrlichia* spp., or *Anaplasma* spp. as determined through pre-enrollment blood tests, poor hair coat, and any disease manifestation that could affect the study outcome. From the 38 dogs, 16 males and 16 females were selected. All were intact, aged approximately 9 months and weighed 6.1–8.8 kg on day − 2. Throughout the study, the dogs were housed in individual concrete pens (except for the first 4 h postinfestation when they were individually confined in travel crates) in a thermostatically controlled environment with an approximate 12-h light/12-h dark cycle. Water was provided ad libitum in bowls, and a commercial ration was provided daily according to manufacturer recommendation. After randomization, contact between study dogs was not allowed.

#### Randomization and treatment

To evaluate susceptibility to experimental infestation, and for randomization, the 38 dogs were each infested on day − 7 with 50 laboratory-reared, unfed, adult *A. americanum* (approximately 50:50 female:male). Counts of live attached ticks were recorded on day − 5 (approximately 48 h postinfestation). The six dogs not meeting inclusion criteria (not accepting gentle restraint to allow tick counting) or having the lowest tick counts were excluded, and the remaining 32 were ordered by descending counts of live attached ticks into eight blocks of four. Within blocks, each dog was randomized to one of four study groups using the randomization function on a spreadsheet program (Microsoft Excel 2019, Redmond, WA, USA). One group remained untreated as a control. The other groups were treated on day 0 with either the combination product of sarolaner, moxidectin, and pyrantel (Simparica Trio™), afoxolaner (NexGard™), or lotilaner (Credelio™). Treatments were administered strictly according to product label, based on body weights recorded on day − 2 using a calibrated weigh scale. All dogs consumed more than 50% of their normal daily ration within 30 min prior to treatment. Dogs were observed for any immediate (within 5 min) reactions to product administration. In addition, general health observations were made at 1 h (± 15 min), 2 h (± 30 min), and 4 h (± 30 min) post-treatment and then daily throughout the study to identify any possible adverse events.

### Tick infestations and counts

Specific pathogen-free, laboratory-reared, adult *A. americanum* ticks purchased from a commercial tick-rearing facility (Ecto Services, Inc., Henderson, NC) were maintained in humidity chambers at ≥ 90% relative humidity until used in the study. The sourcing tick colony is genetically refreshed every 1 to 2 years with *A. americanum* captured from the local area in North Carolina. The ticks were 11–14 weeks post nymph-to-adult molt at study use. For each infestation, 50 adult, unfed, mixed sex (approximately 50:50 male:female) *A. americanum* were deposited along the dorsum (between neck and hips) of manually restrained, unsedated dogs. To facilitate attachment after tick exposure, each dog was individually confined for 4 h in an appropriately sized travel crate. Tick infestations were completed on day − 7 (for determination of each dog’s susceptibility to tick attachment), − 2, 21, and 28.

#### Tick counts

For tick counting, each dog was manually restrained, without sedation, and a thorough body search was conducted for at least 10 min. Any observed tick was classified as male or female, live or dead, and attached or unattached. A tick was classified as dead if it showed no responsive movement, including leg motion when stimulated (breathing on tick and probing with fingers or forceps). All ticks were counted and removed with forceps on day − 5 (for blocking), at 72 h (± 60 min) post-treatment and at 72 h (± 60 min) following each subsequent infestation. In situ ticks from the day − 2 infestation were counted, without removal, at 4 h (± 30 min), 8 h (± 30 min), 12 h (± 30 min), 24 h (± 60 min), and 48 h (± 60 min) after treatments had been administered. The same tick counting timepoints were used following infestations on day 21 and 28.

#### Assessment of unattached ticks collected from crates and dogs.

Following infestation challenges on day 21 and 28, after dogs were removed from crates (4 h postinfestation), both the crate and the dog were inspected for unattached ticks. Any live, unattached ticks found on a dog at 4 h postinfestation were counted, removed from the dog and pooled by study group into vials and placed into a humidified container. Any live free ticks found in crates were similarly collected and stored. Ticks were assessed as live or dead at 12 h (± 2 h), 24 h (± 2 h), and 48 h (± 2 h) after collection from dogs or crates. Any unattached live ticks found on a dog subsequent to the 4-h postinfestation timepoint on day 21 and 28 were counted, recorded as male or female, live or dead, and left in place on the dog.

### Data analysis

Descriptive statistics (geometric mean, arithmetic mean, standard deviation, minimum, median, and maximum values) were calculated for each infestation. Counts of live ticks were analyzed separately for each timepoint of each study infestation, under the linear mixed model. Treatment group was the fixed effect and block the random effect. Error term variance was taken as heterogeneous with respect to treatment group. For efficacy assessments based on geometric means, counts of live ticks were subjected to ln(count + 1) transformation before statistical modeling. Because it is regarded as providing the most appropriate measure of central tendency, statistics presented for the primary objective results (efficacy of lotilaner, sarolaner and afoxolaner at each study timepoint) were based on geometric means [35]. Efficacy of each product was calculated using the formula:

$${\text{Percent efficacy}} = {1}00 \, * \, \left( {{\text{Mc}}{-}{\text{Mt}}} \right)/{\text{Mc}},$$

where *Mc* is the mean number of live ticks on dogs in the untreated control group, and *Mt* is the mean number of live ticks on dogs in a treated group.

Using the same equation, efficacy assessments were also calculated based on arithmetic means with counts of live ticks modeled directly without any transformation (for arithmetic mean calculations see Additional files 1–3: Tables S1–S3). To assure convergence, variances for block and error term were bounded low by 10^–3^ for transformed counts and 10^–1^ for untransformed counts. Pairwise comparisons were carried out using two-sided *t*-tests derived from the statistical model (SAS for Windows, version 9.4, Cary NC, USA). All tests were conducted at the 0.05 significance level. No multiplicity adjustment was performed. To determine if there was a significant decline in acaricidal activity with time after treatment, the day 0 initial speed of kill was compared with day 28 speed of kill at the 12, 24, 48, and 72 h timepoints using the natural log(tick counts + 1). Prior to the analysis, the counts were normalized by subtracting the geometric mean untreated control value at each day and timepoint for each dog. The difference between day 0 and 28 was calculated for each dog and timepoint. Within each product-treated group, differences were assessed by the paired *t*-test or the Wilcoxon signed rank test, depending on the distribution of the data. If the *P* value of the Shapiro–Wilk test was > 0.01 then it was assumed that the data followed a normal distribution and the paired t-test was applied. Otherwise, the Wilcoxon signed rank test was applied.

## Results

### Product dosing and safety

All treatments were well tolerated. Assessment of prestudy blood samples found no evidence of prior exposure to tick-borne pathogens. All products were administered per label per study protocol and there were no events of regurgitation or lost doses. Average study dog weight (± standard deviation) was 7.3 (± 0.80) kg, with the average dog weight in the sarolaner, afoxolaner, lotilaner, and untreated control groups being 7.6 (± 0.76) kg, 7.3 (± 0.83) kg, 7.2 (± 0.87) kg, and 7.2 (± 0.76) kg, respectively. Actual dose ranged from: 1.4–1.8 mg/kg sarolaner; 3.4–4.7 mg/kg afoxolaner; and 26.6–36.9 mg/kg lotilaner. No serious adverse events were observed during the course of the study. One dog in the sarolaner group had bloody stool of < 24 h duration (day 15) that resolved without treatment intervention.

### Tick counts and product efficacy

Following each infestation challenge with 50 *A. americanum*, > 13 live ticks (> 25% of infestation challenge) were found on all untreated control group dogs at every assessment, with counts ranging from 16 to 50. At every timepoint for each infestation challenge, control dogs met the minimum “live attached tick number” requirement (≥ 25% of an infestation challenge) for a valid assessment of the efficacy of the administered products [35, 36].

#### Initial speed of tick kill

At 4 and 8 h post-treatment, all study dogs carried at least 21 live ticks from the day 2 infestation (Table 1). Relative to the untreated group, mean tick count reductions in the lotilaner group were first significant at 12 h post-treatment (*t*_(21)_ = 3.62*; P* = 0.002) (Table 2). Mean live tick counts for dogs in the sarolaner and afoxolaner groups were not significantly different from the control group until 24 h post-treatment. Mean live tick counts in the lotilaner group were significantly lower than in the sarolaner group at 12 (*t*_(21)_ = 2.71; *P* = 0.013), 24 (*t*_(21)_ = 3.48; *P* = 0.002), and 48 h (*t*_(21)_ = 2.38; *P* = 0.027) post-treatment, and afoxolaner at 8 (*t*_(21)_ = 2.28; *P* = 0.033), 12 (*t*_(21)_ = 3.56; *P* = 0.002), 48 (*t*_(21)_ = 2.63; *P* = 0.016), and 72 h (*t*_(21)_ = 4.71; *P* < 0.001). Efficacy ≥ 90%, denoting initial speed of kill, was first achieved in the lotilaner and afoxolaner groups at 24 h post-treatment, and in the sarolaner group at 48 h (Table 2).

**Table 1:** Mean live *Amblyomma americanum* counts from infested dogs, left untreated or following treatment on day 0 with sarolaner, afoxolaner, or lotilaner (8 dogs per group)

	Hours post-treatment	4	8	12	24	48	72
Untreated	Geometric mean (SE)	32.1 (0.1)	32.5 (0.1)	34.1 (0.1)	38.8 (0.3)	37.2 (0.2)	39.9 (0.2)
	Arithmetic mean (SE)	33.1 (2.4)	32.9 (2.1)	34.8 (2.9)	39.3 (2.0)	37.8 (1.5)	40.1 (1.0)
	Median	33.0	33.0	33.0	40.0	38.5	41.5
	Range	22–46	25–42	27–47	30–49	25–47	32–47
Sarolaner (Simparica Trio)	Geometric mean (SE)	33.8 (0.1)	32.3 (0.1)	29.5 (0.1)	10.1 (0.3)	2.0 (0.2)	0.8 (0.2)
	Arithmetic mean (SE)	34.4 (2.4)	32.9 (2.1)	31.3 (2.9)	11.6 (2.0)	3.0 (1.5)	1.3 (1.0)
	Median	34.5	31.5	32.5	12.5	2.0	0.5
	Range	23–42	24–43	17–50	3–20	0–12	0–5
Afoxolaner (NexGard)	Geometric mean (SE)	31.5 (0.1)	34.3 (0.1)	33.7 (0.1)	0.9 (0.3)	2.2 (0.2)	3.2 (0.2)
	Arithmetic mean (SE)	31.8 (2.4)	34.4 (2.1)	34.0 (2.9)	1.5 (2.0)	3.1 (1.5)	3.6 (1.0)
	Median	31.5	33.0	34.0	1.0	2.0	4.0
	Range	25–38	32–40	26–39	0–7	0–9	1–6
Lotilaner (Credelio)	Geometric mean (SE)	30.0 (0.1)	27.8 (0.1)	19.3 (0.1)	1.8 (0.3)	0.6 (0.2)	0.4 (0.2)
	Arithmetic mean (SE)	30.6 (2.4)	28.5 (2.1)	20.8 (2.9)	4.7 (2.0)	0.8 (1.5)	0.5 (1.0)
	Median	28.0	26.5	19.5	1.0	0.5	0.0
	Range	23–42	21–44	8–33	0–20	0–2	0–2

Sarolaner product combined with moxidectin and pyrantel

Infestations made on day-2 with 50 *A. americanum* per dog

SE, standard error

**Table 2:** Efficacy of sarolaner, afoxolaner, and lotilaner at hours following treatment on day 0 of *Amblyomma americanum*-infested dogs (8 dogs per group)

Hours^a^		Sarolaner (Simparica Trio)	Afoxolaner (NexGard)	Lotilaner (Credelio)
4	Efficacy (%)	0.0	1.9	6.6
	Statistics versus untreated	*t*_(21)_ = −0.50*; P* = 0.624	*t*_(21)_ = 0.19*; P* = 0.855	*t*_(21)_ = 0.65*; P* = 0.521
	Statistics versus sarolaner		*t*_(21)_ = 0.68*; P* = 0.502	*t*_(21)_ = 1.15*; P* = 0.263
	Statistics versus afoxolaner			*t*_(21)_ = 0.47*; P* = 0.645
8	Efficacy (%)	0.4	0.0	14.5
	Statistics versus untreated	*t*_(21)_ = 0.05*; P* = 0.962	*t*_(21)_ = −0.59*; P* = 0.563	*t*_(21)_ = 1.69*; P* = 0.105
	Statistics versus sarolaner		*t*_(21)_ = −0.64*; P* = 0.532	*t*_(21)_ = 1.64*; P* = 0.115
	Statistics versus afoxolaner			*t*_(21)_ = 2.28*; P* = 0.033
12	Efficacy (%)	13.3	1.0	43.3
	Statistics versus untreated	*t*_(21)_ = 0.92*; P* = 0.368	*t*_(21)_ = 0.07*; P* = 0.947	*t*_(21)_ = 3.62*; P* = 0.002
	Statistics versus sarolaner		*t*_(21)_ = −0.85*; P* = 0.404	*t*_(21)_ = 2.71*; P* = 0.013
	Statistics versus afoxolaner			*t*_(21)_ = 3.56*; P* = 0.002
24	Efficacy (%)	74.0	97.6	95.3
	Statistics versus untreated	*t*_(21)_ = 3.27*; P* = 0.004	*t*_(21)_ = 7.73*; P* < 0.001	*t*_(21)_ = 6.75*; P* < 0.001
	Statistics versus sarolaner		*t*_(21)_ = 4.46*; P* < 0.001	*t*_(21)_ = 3.48*; P* = 0.002
	Statistics versus afoxolaner			*t*_(21)_ = −0.98*; P* = 0.340
48	Efficacy (%)	94.6	94.0	98.5
	Statistics versus untreated	*t*_(21)_ = 9.28*; P* < 0.001	*t*_(21)_ = 9.03*; P* < 0.001	*t*_(21)_ = 11.66*; P* < 0.001
	Statistics versus sarolaner		*t*_(21)_ = −0.25*; P* = 0.805	*t*_(21)_ = 2.38*; P* = 0.027
	Statistics versus afoxolaner			*t*_(21)_ = 2.63*; P* = 0.016
72	Efficacy (%)	98.1	91.9	99.1
	Statistics versus untreated	*t*_(21)_ = 13.10*; P* < 0.001	*t*_(21)_ = 9.47*; P* < 0.001	*t*_(21)_ = 14.18*; P* < 0.001
	Statistics versus sarolaner		*t*_(21)_ = −3.63*; P* = 0.002	*t*_(21)_ = 1.08*; P* = 0.291
	Statistics versus afoxolaner			*t*_(21)_ = 4.71*; P* < 0.001

Efficacy based on geometric mean live *A. americanum* counts

Sarolaner product combined with moxidectin and pyrantel

^a^Hours post-treatment

Infestations made on day − 2 with 50 *A. americanum* per dog

#### Residual speed of tick kill: infestation challenge 21 days post-treatment

**Fig. 1:**
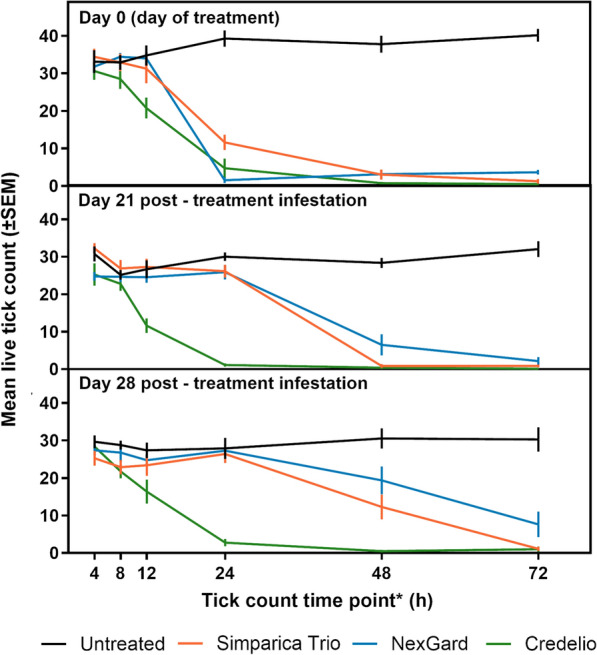
Mean live tick counts ± standard error of the mean (± SEM) of dogs left untreated or treated on day 0 with either Simparica Trio (sarolaner-moxidectin-pyrantel), NexGard (afoxolaner) or Credelio (lotilaner) at a timepoint (h) post-treatment and following infestations on day 21 and 28 (each infestation with 50 *Amblyomma americanum*). *Day 0 counts are relative to time post-treatment; day 21 and 28 counts represent time following infestations

Following *A. americanum* infestations on day 21, mean live tick counts had declined to < 1 in the lotilaner group by 24 h postinfestation, in the sarolaner group by 48 h, but not in the afoxolaner group at any assessment following this infestation (Table 3). Relative to the untreated control group, mean live tick count reductions in the lotilaner group were first significant at 12 h postinfestation (*t*_(21)_ = 5.88; *P* < 0.001), with efficacy exceeding 90% at 24 h (Table 4; Fig. 1). Mean live tick counts in the sarolaner and afoxolaner groups were not significantly lower than in the control group until 48 h postinfestation (*t*_(21)_ = 9.31; *P* < 0.001 and *t*_(21)_ = 6.17; *P* < 0.001, respectively), with efficacy ≥ 90% achieved at 48 h postinfestation in the sarolaner group, and at 72 h in the afoxolaner group.

**Table 3:** Mean live *Amblyomma americanum* counts following a day 21 challenge of dogs left untreated or treated on day 0 with sarolaner, afoxolaner, or lotilaner (8 dogs per group)

	Hours post-challenge	4	8	12	24	48	72
Untreated	Geometric mean (SE)	30.2 (0.1)	24.9 (0.1)	25.8 (0.1)	29.9 (0.1)	28.1 (0.3)	31.5 (0.2)
	Arithmetic mean (SE)	30.8 (2.0)	25.1 (1.8)	26.6 (2.0)	30.0 (1.4)	28.4 (1.6)	32.0 (1.2)
	Median	31.5	26.0	29.5	29.5	28.5	32.0
	Range	20–38	20–30	16–33	27–36	23–33	24–43
Sarolaner (Simparica Trio)	Geometric mean (SE)	32.0 (0.1)	26.2 (0.1)	26.6 (0.1)	25.8 (0.1)	0.5 (0.3)	0.6 (0.2)
	Arithmetic mean (SE)	32.3 (2.0)	26.9 (1.8)	27.3 (2.0)	26.1 (1.4)	0.9 (1.6)	0.9 (1.2)
	Median	32.5	29.5	28.5	25.0	0.0	0.5
	Range	26–40	18–35	18–38	21–36	0–4	0–3
Afoxolaner (NexGard)	Geometric mean (SE)	24.6 (0.1)	24.3 (0.1)	24.2 (0.1)	25.4 (0.1)	3.1 (0.3)	1.2 (0.2)
	Arithmetic mean (SE)	24.8 (2.0)	24.6 (1.8)	24.5 (2.0)	25.9 (1.4)	6.5 (1.6)	2.1 (1.2)
	Median	24.5	25.0	24.5	24.5	4.0	1.0
	Range	20–30	18–29	18–31	20–37	0–23	0–9
Lotilaner (Credelio)	Geometric mean (SE)	23.6 (0.1)	22.2 (0.1)	10.4 (0.1)	0.8 (0.1)	0.2 (0.3)	0.1 (0.2)
	Arithmetic mean (SE)	25.3 (2.0)	22.8 (1.8)	11.6 (2.0)	1.1 (1.4)	0.4 (1.6)	0.1 (1.2)
	Median	29.5	21.0	11.0	0.5	0.0	0.0
	Range	9–32	16–34	4–20	0–3	0–3	0–1

Sarolaner product combined with moxidectin and pyrantel

Infestations with 50 *A. americanum* per dog

SE, standard error

**Table 4:** Efficacy of sarolaner, afoxolaner, and lotilaner to control an *Amblyomma americanum* infestation challenge 21 days post-treatment (8 dogs per group)

Hours^a^		Sarolaner (Simparica Trio)	Afoxolaner (NexGard)	Lotilaner (Credelio)
4	Efficacy (%)	0.0	18.7	21.9
	Statistics versus untreated	*t*_(21)_ = −0.45*; P* = 0.656	*t*_(21)_ = 1.62*; P* = 0.120	*t*_(21)_ = 1.92; *P* = 0.068
	Statistics versus sarolaner		*t*_(21)_ = 2.07*; P* = 0.051	*t*_(21)_ = 2.38*; P* = 0.027
	Statistics versus afoxolaner			*t*_(21)_ = 0.30*; P* = 0.764
8	Efficacy (%)	0.0	2.2	10.6
	Statistics versus untreated	*t*_(21)_ = −0.61*; P* = 0.546	*t*_(21)_ = 0.27*; P* = 0.790	*t*_(21)_ = 1.38*; P* = 0.182
	Statistics versus sarolaner		*t*_(21)_ = 0.88*; P* = 0.387	*t*_(21)_ = 1.99*; P* = 0.060
	Statistics versus afoxolaner			*t*_(21)_ = 1.11*; P* = 0.280
12	Efficacy (%)	0.0	6.3	59.6
	Statistics versus untreated	*t*_(21)_ = −0.21; *P* = 0.835	*t*_(21)_ = 0.43; *P* = 0.671	*t*_(21)_ = 5.88; *P* < 0.001
	Statistics versus sarolaner		*t*_(21)_ = 0.64; *P* = 0.528	*t*_(21)_ = 6.09; *P* < 0.001
	Statistics versus afoxolaner			*t*_(21)_ = 5.45; *P* < 0.001
24	Efficacy (%)	13.6	14.9	97.4
	Statistics versus untreated	*t*_(21)_ = 0.86; *P* = 0.398	*t*_(21)_ = 0.95; *P* = 0.353	*t*_(21)_ = 17.42; *P* < 0.001
	Statistics versus sarolaner		*t*_(21)_ = 0.09; *P* = 0.931	*t*_(21)_ = 16.56; *P* < 0.001
	Statistics versus afoxolaner			*t*_(21)_ = 16.47; *P* < 0.001
48	Efficacy (%)	98.1	88.8	99.3
	Statistics versus untreated	*t*_(21)_ = 9.31; *P* < 0.001	*t*_(21)_ = 6.17; *P* < 0.001	*t*_(21)_ = 10.10; *P* < 0.001
	Statistics versus sarolaner		*t*_(21)_ = −3.14; *P* = 0.005	*t*_(21)_ = 0.80; *P* = 0.435
	Statistics versus afoxolaner			*t*_(21)_ = 3.94; *P* < 0.001
72	Efficacy (%)	98.0	96.1	99.7
	Statistics versus untreated	*t*_(21)_ = 11.36; *P* < 0.001	*t*_(21)_ = 10.16; *P* < 0.001	*t*_(21)_ = 12.86; *P* < 0.001
	Statistics versus sarolaner		*t*_(21)_ = −1.20; *P* = 0.245	*t*_(21)_ = 1.50; *P* = 0.147
	Statistics versus afoxolaner			*t*_(21)_ = 2.70; *P* = 0.013

Efficacy based on geometric mean live *A. americanum* counts

Sarolaner product combined with moxidectin and pyrantel

^a^Hours postinfestation 21-days post-treatment

Infestations with 50 *A. americanum* per dog

#### Residual speed of tick kill: infestation challenge 28 days post-treatment

Following *A. americanum* infestations on day 28, mean live tick counts had declined to < 1 in the lotilaner group by 48 h postinfestation, in the sarolaner group by 72 h, but not in the afoxolaner group at any assessment following this infestation (Table 5). Relative to the untreated control group, mean live tick count reductions in the lotilaner group were first significant at 8 h postinfestation (*t*_(21)_ = 2.64*; P* = 0.015) and remained significantly lower throughout the later time points, with efficacy ≥ 90% at 24 h postinfestation (Table 6). In contrast, the mean live tick counts in the sarolaner group were not consistently significantly lower than the untreated group until 48 h postinfestation. Mean live tick counts in the afoxolaner group were not significantly different from the untreated group until 72 h postinfestation (*t*_(21)_ = 5.09; *P* < 0.001). Speed-of-tick-kill efficacy ≥ 90% was achieved at 72 h in the sarolaner group, when afoxolaner efficacy was 86.3%.

**Table 5:** Mean live *Amblyomma americanum* counts following a day 28 challenge of dogs left untreated or treated on day 0 with sarolaner, afoxolaner or lotilaner (8 dogs per group)

	Hours post-challenge	4	8	12	24	48	72
Untreated	Geometric mean (SE)	29.3 (0.1)	28.6 (0.1)	26.9 (0.1)	26.9 (0.1)	29.7 (0.3)	29.2 (0.3)
	Arithmetic mean (SE)	29.6 (1.9)	28.8 (1.9)	27.4 (2.5)	27.9 (2.0)	30.5 (2.9)	30.3 (2.4)
	Median	30.0	29.5	26.5	28.0	29.0	29.0
	Range	21–35	22–32	19–38	16–41	20–40	19–50
Sarolaner (Simparica Trio)	Geometric mean (SE)	24.7 (0.1)	22.3 (0.1)	22.3 (0.1)	25.6 (0.1)	9.1 (0.3)	0.8 (0.3)
	Arithmetic mean (SE)	25.3 (1.9)	22.9 (1.9)	23.4 (2.5)	26.4 (2.0)	12.3 (2.9)	1.1 (2.4)
	Median	24.0	22.5	20.5	25.5	9.5	1.0
	Range	18–33	16–31	15–35	16–38	2–26	0–5
Afoxolaner (NexGard)	Geometric mean (SE)	27.1 (0.1)	25.9 (0.1)	24.4 (0.1)	27.1 (0.1)	14.0 (0.3)	4.0 (0.3)
	Arithmetic mean (SE)	27.4 (1.9)	26.8 (1.9)	24.8 (2.5)	27.3 (2.0)	19.4 (2.9)	7.6 (2.4)
	Median	26.5	26.5	24.0	27.5	20.0	3.0
	Range	23–36	17–37	20–33	24–32	0–34	0–26
Lotilaner (Credelio)	Geometric mean (SE)	27.8 (0.1)	21.3 (0.1)	14.0 (0.1)	2.1 (0.1)	0.3 (0.3)	0.5 (0.3)
	Arithmetic mean (SE)	28.4 (1.9)	21.8 (1.9)	16.4 (2.5)	2.8 (2.0)	0.5 (2.9)	1.0 (2.4)
	Median	27.0	21.5	15.5	2.5	0.0	0.0
	Range	22–38	14–32	5–29	0–9	0–3	0–6

Sarolaner product combined with moxidectin and pyrantel

Infestations with 50 *A. americanum* per dog

SE, standard error

**Table 6:** Efficacy of sarolaner, afoxolaner and lotilaner to control an *Amblyomma americanum* infestation challenge 28 days post-treatment (8 dogs per group)

Hours^a^		Sarolaner (Simparica Trio)	Afoxolaner (NexGard)	Lotilaner (Credelio)
4	Efficacy (%)	15.5	7.4	4.9
	Statistics versus untreated	*t*_(21)_ = 1.76*; P* = 0.093	*t*_(21)_ = 0.80*; P* = 0.432	*t*_(21)_ = 0.53; *P* = 0.601
	Statistics versus sarolaner		*t*_(21)_ = −0.96*; P* = 0.348	*t*_(21)_ = −1.23*; P* = 0.232
	Statistics versus afoxolaner			*t*_(21)_ = −0.27*; P* = 0.789
8	Efficacy (%)	21.8	9.2	25.6
	Statistics versus untreated	*t*_(21)_ = 2.21*; P* = 0.039	*t*_(21)_ = 0.87*; P* = 0.396	*t*_(21)_ = 2.64*; P* = 0.015
	Statistics versus sarolaner		*t*_(21)_ = −1.34*; P* = 0.195	*t*_(21)_ = 0.44*; P* = 0.666
	Statistics versus afoxolaner			*t*_(21)_ = 1.78*; P* = 0.090
12	Efficacy (%)	17.1	9.0	47.8
	Statistics versus untreated	*t*_(21)_ = 1.04; *P* = 0.311	*t*_(21)_ = 0.52; *P* = 0.607	*t*_(21)_ = 3.56; *P* = 0.002
	Statistics versus sarolaner		*t*_(21)_ = −0.52; *P* = 0.612	*t*_(21)_ = 2.52; *P* = 0.020
	Statistics versus afoxolaner			*t*_(21)_ = 3.04; *P* = 0.006
24	Efficacy (%)	4.9	0.0	92.3
	Statistics vs untreated	*t*_(21)_ = 0.27; *P* = 0.788	*t*_(21)_ = −0.04; *P* = 0.965	*t*_(21)_ = 12.54; *P* < 0.001
	Statistics versus sarolaner		*t*_(21)_ = −0.32; *P* = 0.754	*t*_(21)_ = 12.27; *P* < 0.001
	Statistics versus afoxolaner			*t*_(21)_ = 12.59; *P* < 0.001
48	Efficacy (%)	69.3	53.0	99.0
	Statistics versus untreated	*t*_(21)_ = 3.12; *P* = 0.005	*t*_(21)_ = 2.01; *P* = 0.057	*t*_(21)_ = 8.88; *P* < 0.001
	Statistics versus sarolaner		*t*_(21)_ = −1.10; *P* = 0.282	*t*_(21)_ = 5.76; *P* < 0.001
	Statistics versus afoxolaner			*t*_(21)_ = 6.86; *P* < 0.001
72	Efficacy (%)	97.4	86.3	98.2
	Statistics versus untreated	*t*_(21)_ = 8.02; *P* < 0.001	*t*_(21)_ = 5.09; *P* < 0.001	*t*_(21)_ = 8.46; *P* < 0.001
	Statistics versus sarolaner		*t*_(21)_ = −2.94; *P* = 0.008	*t*_(21)_ = 0.44; *P* = 0.668
	Statistics versus afoxolaner			*t*_(21)_ = 3.37; *P* = 0.003

Efficacy based on geometric mean live *A. americanum* counts

Sarolaner product combined with moxidectin and pyrantel

Infestations with 50 *A. americanum* per dog

^a^Hours postinfestation 28-days post-treatment

Following normalization of live tick counts to the untreated group, comparison of counts within the sarolaner and afoxolaner groups detected significantly slower speed-of-kill on day 28 compared with post-treatment on day 0. Mean live tick counts in the sarolaner group were significantly greater at 24 (*P* = 0.002) and 48 h (*P* = 0.005) after the day 28 infestation compared with the equivalent day 0 post-treatment timepoints. For afoxolaner, mean live tick counts were significantly greater at 24 (*P* < 0.001) and 48 h (*P* < 0.001) after the day 28 infestation compared with the equivalent day 0 post-treatment timepoints. In contrast, for lotilaner, mean live tick counts were similarly low at 24 (*P* = 0.405) and 48 h (*P* = 0.882) after the infestation on day 28 compared with the equivalent day 0 post-treatment timepoints.

## Discussion

A rapid speed of tick kill of canine acaricidal products is critical, because the longer a tick feeds, the greater the risk of pathogen transmission [20]. Speed of tick kill can be linked to parasite susceptibility to the drug, drug dose, how rapidly that dose is absorbed to reach systemically active blood levels, and the rate of drug elimination.

Following the first oral administration to dogs, peak blood levels are documented to occur within 2 h for lotilaner, within 2–6 h for afoxolaner, and within 3.5 h for sarolaner in its combination product [37–39]. Consistent with rapid product absorption, in this study the speed of *A. americanum* kill at 24 h post-treatment with either afoxolaner (efficacy 97.6%) or lotilaner (95.3%) was faster than that of the sarolaner combination product (74.0%). An earlier study showed that a sarolaner dose of 2–4 mg/kg (label dose of the single entity product, Simparica™) provided 100% efficacy at 24 h post-treatment of an existing *A. americanum* infestation [40]. It appears that the lower sarolaner dose in the combination product (minimum 1.2 mg/kg; range 1.4–1.8 mg/kg in this study) results in a slower initial speed of tick kill than the dose in the single entity sarolaner product.

Sustainability of the speed of tick kill, important in maintaining a reduced risk of tick-borne pathogen transmission, may be influenced by different rates of declining drug levels throughout the labeled treatment period. The longer half-life of lotilaner following oral administration (30.7 days), compared with sarolaner (12 days) and afoxolaner (12.8–15.5 days), may allow the more rapid speed of tick kill for lotilaner to be sustained for a longer duration than with either the combination sarolaner product or afoxolaner [37–39]. Evidence corroborating that suggestion comes from the *A. americanum* infestation challenges on days 21 and 28. At 12 h after the day 21 challenge, lotilaner efficacy against *A. americanum* was significantly greater than either competitor product, and was > 90% at 24 h, while sarolaner and afoxolaner did not achieve that level of efficacy until 48 and 72 h post-challenge, respectively. Similarly, at 24 h after the day 28 challenge, lotilaner efficacy was again > 90%, while sarolaner did not achieve that speed of tick kill until 72 h post-challenge. At no point in the study was there a significantly lower mean live tick count in either the sarolaner or afoxolaner groups compared with the lotilaner group.

Within each product-treated group, the speed of tick kill on day 28 was compared with that on day 0. At all assessed timepoints lotilaner was shown to sustain equivalent speed of kill between day 0 and day 28. This was not the case for the sarolaner combination product and afoxolaner, for which a reduced speed of kill on day 28, relative to day 0, was present at the 24 and 48 h timepoints. For sarolaner, this finding aligns with earlier reports suggesting that, relative to the stand-alone sarolaner product (Simparica, 2.0 mg/kg), the 40% reduction in minimum dose of the combination (Simparica Trio; 1.2 mg/kg) results in slower residual tick-killing activity [34, 40]. Of note, the sarolaner combination product was chosen for this study due to its relative market prominence. A combination product for dogs containing afoxolaner (at the same dose rate as used in this study), moxidectin and pyrantel was not available at the time of this study.

Inconsistent with two USA registration studies, in the current study afoxolaner failed to achieve 90% efficacy within 72 h following the day 28 infestation challenge [41]. At the final assessment, just one afoxolaner-treated dog was free of ticks, and two dogs had live attached tick counts of 19 and 26. It would therefore appear that, both in this population of dogs and in dogs enrolled in an earlier study, there was a substantial end-of-month decline in afoxolaner efficacy against *A. americanum* [40]. The normal appearance of the live attached ticks taken from afoxolaner-treated dogs at 72 h postinfestation on day 28 is indicative of viability. Those ticks could have potential to produce eggs and so continue the life cycle, or to find and infest a second host. In contrast, live attached ticks taken from lotilaner-treated dogs at the same timepoint were moribund, and so unlikely to continue feeding or infest a second host. This finding is supportive of earlier results showing that live attached ticks removed from lotilaner-treated dogs will die soon after detachment [42].

A value-added aspect of this study is the fact that three USA market-leading monthly isoxazoline-containing parasiticide products were directly compared in a head-to-head manner, eliminating the inherent variability of comparing results across studies. Extrapolation of these results is also relevant to other isoxazoline speed-of-tick-kill studies. The single lotilaner speed-of-kill report described infestations of dogs with *Ixodes ricinus.* Efficacy was 99.2% at 8 h post-treatment and 97.5% at 12 h after a day 28 challenge [43]. The 76.7% *I. ricinus* efficacy shown for the single-entity sarolaner product at 8 h post-treatment was lower than that reported for lotilaner, although at 12 h sarolaner efficacy (94.9%) was similar to that of lotilaner (98.0%) [43, 44]. Against *I. scapularis*, efficacy of that sarolaner product at 12 h after a day 28 challenge was 62.0% [44]. Two reports have described the speed-of-kill of the combination sarolaner product (used in the current study) against induced infestations with *I. scapularis*. In one study, efficacy at 12 and 24 h declined from 98.4 and 99.4% immediately following treatment, respectively, to 52.2 and 94.2% on day 28 [45]. In the other study, the 12- and 24-h post-treatment efficacies were 93.0% and 99.5%, respectively, declining to 27.7% and 96.6% following a day 28 challenge [33]. The authors suggested that the lower sarolaner dose results in a significantly slower residual speed of kill of *I. scapularis*. In a previous study for afoxolaner, efficacy against *I. scapularis* declined from 99.5% at 24 h post-treatment to 71.8% at 24 h after a day 28 challenge [34]. Taken with the results of the current study, these reports suggest that the speed of tick kill, and overall efficacy, of afoxolaner and the combination sarolaner product decline with time during the treatment period. Conversely, the results also indicate that there is no efficacy decline within lotilaner’s monthly dosing period. Thus, lotilaner’s sustained fast speed of kill, relative to afoxolaner and the combination sarolaner product, may be broadly applicable to ixodid ticks.

Although not a direct objective of this study, the percent of dogs free of live ticks was compared between product-treated groups. At all timepoints, 24, 48, and 72 h post-treatment and following each subsequent infestation, more dogs in the lotilaner group were free of live ticks (tick free) than in either of the other treated groups. At 72 h post-treatment, significantly more dogs in the lotilaner group (5/8) were tick free, relative to the afoxolaner group (*P* = 0.026; 0/8 dogs tick free). At 48 h following the day 28 infestation, significantly more lotilaner-group dogs (6/8) were tick free, relative to the sarolaner (*P* = 0.007; 0/8 tick free) and afoxolaner (1/8) (*P* = 0.041) groups.

Because isoxazoline-containing parasiticide products act systemically, it would be expected that ticks must begin engaging in the attachment process to be exposed to the drug. In contrast with that expectation, an earlier study with lotilaner demonstrated reduced tick attachment to lotilaner-treated dogs compared with untreated control dogs suggesting that ticks (*A. americanum*) may be adversely affected by lotilaner prior to complete attachment [42]. In the present study, no difference in the *A. americanum* attachment rate was noted between any of the product-treated groups or the untreated controls. Furthermore, evaluation of unattached ticks collected from cages and dogs at 4 h following infestations on days 21 and 28 remained lively and viable following incubation for 24 h. The lower *A. americanum* attachment rate observed in the earlier study on lotilaner treated dogs may have been a product of the experimental design, including the preparation of the infestation site (i.e., shaved skin with ticks under bandages) or infestation challenge timing (i.e. 7 days post-treatment).

## Conclusions

This study demonstrates that lotilaner (Credelio) is more efficacious than afoxolaner (NexGard) and the combination of sarolaner–moxidectin–pyrantel (Simparica Trio) in quickly controlling an *A. americanum* infestation. The results show that lotilaner sustains its speed of tick kill advantage throughout the month-long treatment period. In contrast, the speed of kill of the afoxolaner and combination sarolaner products declines between day 0 and 28. Lotilaner’s rapid and consistent speed of kill can be beneficial in reducing the risk of tick-borne pathogen transmission and improving pet owner experience.

### Supplementary Information

Additional file 1: Table S1. Percentage efficacy (based on arithmetic means) of sarolaner, afoxolaner, and lotilaner at hours following treatment of *Amblyomma americanum* infestations on day 0 (*n* = 8 dogs per group).

Additional file 2: Table S2. Percentage efficacy based (based on arithmetic means) against *Amblyomma americanum* of day 0 treatment of dogs with sarolaner, afoxolaner, or lotilaner, compared with untreated control dogs, following challenge on day 21 (*n* = 8 dogs per group).

Additional file 1: Table S3. Percentage efficacy (based on arithmetic means) against *Amblyomma americanum* of day 0 treatment of dogs with sarolaner, afoxolaner, or lotilaner, compared with untreated control dogs, following challenge on day 28 (*n* = 8 dogs per group).

## Data Availability

Data is provided within the manuscript or supplementary information files.
