# Supplementary material for: Comparative speed of kill provided by lotilaner (Credelio™), sarolaner (Simparica Trio™), and afoxolaner (NexGard™) to control Amblyomma americanum infestations on dogs
Source: Parasit Vectors. 2024 Jul 20;17:313. doi: 10.1186/s13071-024-06363-w (PMC11264992; doi:10.1186/s13071-024-06363-w)
Supplement: Supplementary file 1 — Additional file 1: Table S1. Percentage efficacy (based on arithmetic means) of sarolaner, afoxolaner, and lotilaner at hours following treatment of Amblyomma americanum infestations on day 0 (n = 8 dogs per group). [file 13071_2024_6363_MOESM1_ESM.docx]

**Supplementary Table 1.** Percentage efficacy of sarolaner, afoxolaner and lotilaner, based on arithmetic means, following treatment of *Amblyomma americanum* infestations on Day 0 (n = 8 dogs per group)

| Hours^a^ |  | Sarolaner^b^ | Afoxalaner | Lotilaner |
| --- | --- | --- | --- | --- |
| 4 | Efficacy | 0.0 | 4.2 | 7.5 |
|  | Statistics vs control | *t*_21_ *= -*0.37*; P* = 0.712 | *t*_21_ *=*0 41*; P* = 0.684 | *t*_21_ *=* 0 75*; P* = 0.462 |
|  | Statistics vs sarolaner |  | *t*_21_ *=* 0.79*; P* = 0.440 | *t*_21_ *=* 1 12*; P* = 0.274 |
|  | Statistics vs afoxolaner |  |  | *t*_21_ = 0.34*; P* = 0.739 |
| 8 | Efficacy | 0.0 | 0.0 | 13.3 |
|  | Statistics vs control | *t*_21_ *=* 0.00*; P* = 1.000 | *t*_21_ *=* -0.51*; P* = 0.617 | *t*_21_ *=* 1.48*; P* = 0.154 |
|  | Statistics vs sarolaner |  | *t*_21_ = -0.51*; P* = 0.617 | *t*_21_ = 1.48*; P* = 0.154 |
|  | Statistics vs afoxolaner |  |  | *t*_21_ = 1.99*; P* = 0.060 |
| 12 | Efficacy | 10.1 | 2.2 | 40.3 |
|  | Statistics vs control | *t*_21_ = 0.92*; P* = 0.368 | *t*_21_ = 0.18*; P* = 0.855 | *t*_21_ = 3.45*; P* = 0.002 |
|  | Statistics vs sarolaner |  | *t*_21_ = -0.68*; P* = 0.506 | *t*_21_ *=* 2.58*; P* = 0.017 |
|  | Statistics vs afoxolaner |  |  | *t*_21_ = 3.26*; P* = 0.004 |
| 24 | Efficacy | 70.4 | 97.6 (96.2) | 95.3 (87.9) |
|  | Statistics vs control | *t*_21_ = 9.80*; P* < 0.001 | *t*_21_ *=* 13.39*; P* < 0.001 | *t*_21_ *=* 6.75*; P* < 0.001 |
|  | Statistics vs sarolaner |  | *t*_21_ = 3.59*; P* = 0.002 | *t*_21_ = 2.44*; P* = 0.024 |
|  | Statistics vs afoxolaner |  |  | *t*_21_ = -1.15*; P* = 0.262 |
| 48 | Efficacy | 92.1 | 91.7 | 98.0 |
|  | Statistics vs control | *t*_21_ = 18.92*; P* < 0.001 | *t*_21_ = 18.85*; P* < 0.001 | *t*_21_ = 20.14*; P* < 0.001 |
|  | Statistics vs sarolaner |  | *t*_21_ = -0.07*; P* = 0.946 | *t*_21_ = 1.22*; P* = 0.234 |
|  | Statistics vs afoxolaner |  |  | *t*_21_ = 1.29*; P* = 0.210 |
| 72 | Efficacy | 96.9 | 91.0 | 98.8 |
|  | Statistics vs control | *t*_21_ = 27.85*; P* < 0.001 | *t*_21_ = 26.15*; P* < 0.001 | *t*_21_ = 28.39*; P* < 0.001 |
|  | Statistics vs sarolaner |  | *t*_21_ = -1.70*; P* = 0.104 | *t*_21_ = 0.54*; P* = 0.597 |
|  | Statistics vs afoxolaner |  |  | *t*_21_ = 2.24*; P* = 0.036 |

^a^Hours post treatment ^b^Sarolaner product combined with moxidectin and pyrantel
