# Supplementary material for: Comparative speed of kill provided by lotilaner (Credelio™), sarolaner (Simparica Trio™), and afoxolaner (NexGard™) to control Amblyomma americanum infestations on dogs
Source: Parasit Vectors. 2024 Jul 20;17:313. doi: 10.1186/s13071-024-06363-w (PMC11264992; doi:10.1186/s13071-024-06363-w)
Supplement: Supplementary file 2 — Additional file 2: Table S2. Percentage efficacy based (based on arithmetic means) against Amblyomma americanum of day 0 treatment of dogs with sarolaner, afoxolaner, or lotilaner, compared with untreated control dogs, following challenge on day 21 (n = 8 dogs per group). [file 13071_2024_6363_MOESM2_ESM.docx]

**Supplementary Table 2.** Efficacy (based on arithmetic means) against *Amblyomma americanum* of Day 0 treatments of dogs with sarolaner, afoxolaner or lotilaner, compared with untreated control dogs, following challenge on Day 21 (n = 8 dogs per group)

| Hours^a^ |  | Sarolaner^b^ | Afoxalaner | Lotilaner |
| --- | --- | --- | --- | --- |
| 4 | Efficacy | 0.0 | 19.5 | 19.5 |
|  | Statistics vs control | *t*_21_ *=* -0.52*; P* = 0.605 | *t*_21_ *=* 2.10*; P* = 0.048 | *t*_21_ = 1.92; *P* = 0.068 |
|  | Statistics vs sarolaner |  | *t*_21_ = 2.62*; P* = 0.016 | *t*_21_ *=* 2.45*; P* = 0.023 |
|  | Statistics vs afoxolaner |  |  | *t*_21_ *=* -0.17*; P* = 0.863 |
| 8 | Efficacy | 0.0 | 2.0 | 9.5 |
|  | Statistics vs control | *t*_21_ = -0.86*; P* = 0.398 | *t*_21_ = 0.25*; P* = 0.808 | *t*_21_ = 1.17*; P* = 0.255 |
|  | Statistics vs sarolaner |  | *t*_21_ = 1.11*; P* = 0.280 | *t*_21_ = 2.03*; P* = 0.055 |
|  | Statistics vs afoxolaner |  |  | *t*_21_ = 0.92*; P* = 0.366 |
| 12 | Efficacy | 0.0 | 8.0 | 56.3 |
|  | Statistics vs control | *t*_21_ = -0.23; *P* = 0.824 | *t*_21_ = 0.77; *P* = 0.451 | *t*_21_ = 5.42; *P* < 0.001 |
|  | Statistics vs sarolaner |  | *t*_21_ = 0.99; *P* = 0.332 | *t*_21_ = 5.64; *P* < 0.001 |
|  | Statistics vs afoxolaner |  |  | *t*_28_ = 4.65; *P* < 0.001 |
| 24 | Efficacy | 12.9 | 13.8 | 96.3 |
|  | Statistics vs control | *t*_21_ = 1.91; *P* = 0.069 | *t*_21_ = 2.04; *P* = 0.054 | *t*_21_ = 14.26; *P* < 0.001 |
|  | Statistics vs sarolaner |  | *t*_21_ = 0.12; *P* = 0.903 | *t*_21_ = 12.35; *P* < 0.001 |
|  | Statistics vs afoxolaner |  |  | *t*_28_ = 12.22; *P* < 0.001 |
| 48 | Efficacy | 96.9 | 77.1 | 98.7 |
|  | Statistics vs control | *t*_21_ = 13.51; *P* < 0.001 | *t*_21_ = 10.75; *P* < 0.001 | *t*_21_ = 13.76; *P* < 0.001 |
|  | Statistics vs sarolaner |  | *t*_21_ = -2.76; *P* = 0.012 | *t*_21_ = 0.25; *P* = 0.808 |
|  | Statistics vs afoxolaner |  |  | *t*_21_ = 3.01; *P* = 0.007 |
| 72 | Efficacy | 97.3 | 93.4 | 99.6 |
|  | Statistics vs control | *t*_21_ = 18.12; *P* < 0.001 | *t*_21_ = 17.39; *P* < 0.001 | *t_2_*_1_ = 18.55; *P* < 0.001 |
|  | Statistics vs sarolaner |  | *t*_21_ = -0.73; *P* = 0.475 | *t*_21_ = 0.44; *P* = 0.667 |
|  | Statistics vs afoxolaner |  |  | *t*_21_ = 1.16; *P* = 0.257 |

^a^Hours post treatment ^b^Sarolaner product combined with moxidectin and pyrantel
