# Supplementary material for: Comparative speed of kill provided by lotilaner (Credelio™), sarolaner (Simparica Trio™), and afoxolaner (NexGard™) to control Amblyomma americanum infestations on dogs
Source: Parasit Vectors. 2024 Jul 20;17:313. doi: 10.1186/s13071-024-06363-w (PMC11264992; doi:10.1186/s13071-024-06363-w)
Supplement: Supplementary file 3 — Additional file 1: Table S3. Percentage efficacy (based on arithmetic means) against Amblyomma americanum of day 0 treatment of dogs with sarolaner, afoxolaner, or lotilaner, compared with untreated control dogs, following challenge on day 28 (n = 8 dogs per group). [file 13071_2024_6363_MOESM3_ESM.docx]

**Supplementary Table 3.** Efficacy (based on arithmetic means) against *Amblyomma americanum* of Day 0 treatments of dogs with sarolaner, afoxolaner or lotilaner, compared with untreated control dogs, following challenge on Day 28 (n = 8 dogs per group)

| Hours |  | Sarolaner^b^ | Afoxalaner | Lotilaner |
| --- | --- | --- | --- | --- |
| 4 | Efficacy | 14.8 | 7.6 | 4.2 |
|  | Statistics vs control | *t*_21_ *=* 1.65*; P* = 0.114 | *t*_21_ *=* 0.85*; P* = 0.406 | *t*_21_ = 0.47; *P* = 0.642 |
|  | Statistics vs sarolaner |  | *t*_21_ = -0.80*; P* = 0.432 | *t*_21_ *=* -1.18*; P* = 0.252 |
|  | Statistics vs afoxolaner |  |  | *t*_28_ *=* -0.38*; P* = 0.710 |
| 8 | Efficacy | 20.4 | 7.0 | 24.3 |
|  | Statistics vs control | *t*_21_ = 2.18*; P* = 0.041 | *t*_21_ = 0.74*; P* = 0.467 | *t*_21_ = 2.59*; P* = 0.017 |
|  | Statistics vs sarolaner |  | *t*_21_ = -1.44*; P* = 0.166 | *t*_21_ = 0.42*; P* = 0.681 |
|  | Statistics vs afoxolaner |  |  | *t*_21_ = 1.85*; P* = 0.078 |
| 12 | Efficacy | 14.6 | 9.6 | 40.2 |
|  | Statistics vs control | *t*_21_ = 1.21; *P* = 0.241 | *t*_21_ = 0.79; *P* = 0.437 | *t*_21_ = 3.32; *P* = 0.003 |
|  | Statistics vs sarolaner |  | *t*_21_ = -0.42; *P* = 0.682 | *t*_21_ = 2.11; *P* = 0.047 |
|  | Statistics vs afoxolaner | *t*_21_ = -0.52; *P* = 0.612 |  | *t*_21_ = 2.53; *P* = 0.020 |
| 24 | Efficacy | 5.4 | 2.2 | 90.1 |
|  | Statistics vs control | *t*_21_ = 0.60; *P* = 0.556 | *t*_21_ = -0.25; *P* = 0.806 | *t*_21_ = 10.02; *P* < 0.001 |
|  | Statistics vs sarolaner |  | *t*_21_ = -0.35; *P* = 0.731 | *t*_21_ = 9.42; *P* < 0.001 |
|  | Statistics vs afoxolaner |  |  | *t*_21_ = 9.77; *P* < 0.001 |
| 48 | Efficacy | 59.8 | 36.5 | 98.4 |
|  | Statistics vs control | *t*_21_ = 4.52; *P* < 0.001 | *t*_21_ = 2.75; *P* = 0.012 | *t*_21_ = 7.42; *P* < 0.001 |
|  | Statistics vs sarolaner |  | *t*_21_ = -1.76; *P* = 0.092 | *t*_21_ = 2.91; *P* = 0.008 |
|  | Statistics vs afoxolaner |  |  | *t*_21_ = 4.67; *P* < 0.001 |
| 72 | Efficacy | 96.3 | 74.8 | 96.7 |
|  | Statistics vs control | *t*_21_ = 9.46; *P* < 0.001 | t_21_ = 7.35; *P* < 0.001 | *t*_21_ = 9.50; *P* < 0.001 |
|  | Statistics vs sarolaner |  | *t*_21_ = -2.11; *P* = 0.047 | *t*_21_ = 0.04; *P* = 0.968 |
|  | Statistics vs afoxolaner |  |  | *t*_21_ = 2.15; *P* = 0.043 |

GM geometric mean; AM arithmetic mean ^a^Hours post infestation ^b^Sarolaner product combined with moxidectin and pyrantel
